# Editorial: Management of PJI/SSI after joint arthroplasty

**DOI:** 10.1186/s42836-024-00256-0

**Published:** 2024-06-06

**Authors:** Li Cao, Javad Parvizi, Xiaogang Zhang, Xianzhe Liu, Wierd P. Zijlstra, Saad Tarabichi

**Affiliations:** 1 Department of Orthopedics, the First Affiliated Hospital of Xinjiang Medical University, Urumqi, 830011 China; 2 International Joint Center, Acibadem University Hospital, Istanbul, 34746 Turkey; 3 Department of Orthopedic Surgery, Wuhan Union Hospital, Tongji Medical College, Huazhong University of Science and Technology, Wuhan, 430022 China; 4 Department of Orthopedic Surgery, Medical Center Leeuwarden, 8934 AD Leeuwarden, the Netherlands; 5 Rothman Orthopedic Institute at Thomas Jefferson University Hospital, Philadelphia, PA 19107 USA

**Keywords:** Periprosthetic joint infection, Surgical site infection, Preventive strategy, Diagnostic method, Joint arthroplasty

## Abstract

The management of periprosthetic joint infection (PJI) and surgical site infection (SSI) after joint arthroplasty poses a major challenge in orthopedic surgery. This Editorial provides an overview of the studies published in the special issue “Management of PJI/SSI after Joint Arthroplasty”, summarizing the key findings from these studies, which cover a wide range of topics, including stringent preventive strategies, comprehensive diagnostic methods, and personalized treatment modalities. The authors concluded the editorial with their perspectives regarding the status quo of research in this field and future directions for research, such as the development of novel antibiotics, biofilm research, patient-specific risk factors, and the integration of technological advancements (such as machine learning and artificial intelligence) into clinical practice. The authors emphasized the need for continued research, interdisciplinary collaboration, and the application of innovative technologies to enhance patient outcomes and mitigate the burden of these infections on healthcare systems.

## Introduction

Periprosthetic joint infection (PJI) and surgical site infection (SSI) following joint arthroplasty still present significant challenges in the field of orthopedic surgery. These infections can lead to severe consequences, including protracted hospital stays, increased healthcare costs, patient discomfort, and even implant failure [1, 2]. The management of PJI/SSI requires a comprehensive and multidisciplinary approach to effectively prevent, diagnose, and treat these infections.

Prevention of PJI/SSI begins with strict adherence to infection control guidelines during the perioperative period. Strategies such as preoperative screening for infection risk factors, appropriate surgical site preparation, administration of prophylactic antibiotics, and rigorous sterile technique are critical in reducing the incidence of these infections [3]. However, PJI/SSI can still occur despite stringent preventative measures, so prompt and accurate diagnosis is essential. The diagnosis of PJI/SSI is often complicated and tends to involve clinical assessment, laboratory tests, imaging examinations, and sometimes invasive procedures such as joint aspiration or tissue sampling [4]. Differentiation between aseptic failure and infection is critical to the selection of the appropriate management approach [5]. Various diagnostic criteria and scoring systems have been developed to aid in this process, but challenges remain.

Once diagnosed, the management of PJI/SSI requires an approach tailored to the specific features of the infection, including the causative microorganisms, the extent of tissue involvement, and the stability of the implant [6]. Treatment options range from antimicrobial therapy alone for early and low-grade infections [7] to a combination of various surgical interventions for more severe cases [8]. The choice of treatment modality must carefully consider factors such as patient comorbidities, implant stability, and the potential for functional restoration. Recent years have witnessed significant advances in the understanding and management of PJI/SSI, with research focusing on improving diagnostic accuracy [9], developing novel antimicrobial strategies [10], exploring the role of biofilms in infection persistence [11], and investigating the impact of implant design and surface modifications on infection rates [12].

This special issue “Management of PJI/SSI after Joint Arthroplasty” (Available via: https://www.biomedcentral.com/collections/pji, Accessed on 1 March 2024), is important to the field of PJI/SSI management. It brings together a collection of research papers that addressed the challenges and explored potential solutions associated with these infections. The papers included in this special issue covered various aspects of PJI/SSI, such as advances in diagnostic techniques, innovative treatment modalities, and infection prevention strategies. We aimed to stimulate discussion, generate new ideas, and encourage collaboration between experts in the field, thereby contributing to the ongoing efforts to combat post- arthroplasty PJI/SSI.

### Preventive strategies in joint arthroplasty

SSI prevention is a key aspect of joint replacement management. Parvizi et al. [13] proposed a comprehensive ten-step approach to SSI prevention, involving all stages of the surgical procedure from preoperative planning, including patient education and skin preparation, through intraoperative strategies such as antibiotic prophylaxis and aseptic techniques, to postoperative wound care. They believed that, by consistently following these steps, the risk of SSI could be significantly reduced, thereby enhancing patient safety, and improving surgical outcomes.

The emphasis on prevention is further underscored by some studies that aimed at identifying and managing risk factors associated with SSIs in joint replacement surgery. For example, Lin et al. [14] conducted a study on the incidence and risk factors of SSI following primary hip hemiarthroplasty in elderly patients, identifying chronic steroid use, increased BMI, and prolonged intraoperative time as independent risk factors. They highlighted the importance of tailored interventions to manage these risk factors in this population. Similarly, Chan et al. [15] reviewed the risk factors associated with SSI after joint replacement surgery, with the following patient-related factors taken into account, such as age, BMI, and comorbidities, and surgery-related factors such as surgical technique, duration of surgery, and perioperative management. They emphasized the significance of understanding these risk factors in developing effective prevention strategies. In a similar vein, van de Kuit et al. [16] conducted a systematic review and meta-analysis comparing the risk of SSI in patients undergoing elective knee and hip arthroplasty with either staples or sutures for wound closure. Results indicated that suturing may be a safer choice than stapling (especially in hips), a conclusion that has important implications for surgical practice and patient outcomes in orthopedic surgery. Taken together, these studies highlighted the importance of personalized and evidence-based approaches to reducing the risk of SSI and underscored the critical role of prevention in improving patient safety and surgical outcomes.

Antibiotic-loaded bone cement (ALBC) has been recognized as an important tool in the prevention and treatment of PJI. Soriano et al. [17] examined the use of ALBC and discussed its pharmacokinetics, efficacy in infection prevention, prophylactic and therapeutic effects, and potential risks of antimicrobial resistance. They highlighted the need for careful patient selection and appropriate antibiotic choice to maximize benefits and minimize risks. Berberich et al. [18] reviewed the potential of dual antibiotic-loaded bone cement (ALBC) in the prevention of PJI in high-risk patients. Studies indicated that the local delivery of antibiotics via bone cement could provide enhanced and sustained antimicrobial effects, thereby lowering the incidence of PJI. This paper provided a compelling argument for the potential of ALBC in PJI prevention, especially in high-risk patient groups. However, Bos et al. [19] were unable to prove that dual ALBC was superior to single ALBC in aseptic revision hip and knee surgery.

In addition to these strategies, Zhou et al. [20] stressed the critical role of soft tissue management in the treatment of PJI. They provided an in-depth review of guidelines for soft tissue management at various stages of the surgical process, including preoperative evaluation, surgical exposure, intraoperative removal of infected tissues, defect coverage, postoperative assessment, wound management, and rehabilitation. One of the fundamental aspects was the importance of thorough debridement and reconstruction of soft tissues with a good blood supply for successful PJI treatment. They emphasized that, by carefully following these principles, surgeons could significantly enhance infection control rates and postoperative joint function, leading to improved patient outcomes.

### Diagnostic methods for joint infection

The diagnosis of chronic PJI remains a formidable challenge due to the lack of a “gold standard”. Jennings et al. [21] addressed this complicated issue head-on, emphasizing the need for a comprehensive clinical evaluation, which includes patient history, physical examination, laboratory tests, and imaging studies, for the accurate diagnosis of chronic PJI. They also delved into the possibility of utilizing joint aspiration and the interpretation of multiple diagnostic tests to improve diagnostic accuracy. In addition, they emphasized the need for meticulous assessment of multiple factors and the application of validated scoring systems or consensus-based criteria for PJI diagnosis, thereby providing valuable guidance that helps clinicians make informed decisions. This comprehensive approach confronts the challenges of diagnosing chronic PJI and highlights the importance of integrating different diagnostic modalities and patient-specific factors into the diagnostic workup.

Advances in diagnostic techniques and biomarkers have significant potential to improve the accuracy of PJI diagnosis. This was the focus of a study by Tripathi et al. [22], who explored the potential of these advancements. They studied the use of novel biomarkers in conjunction with traditional biomarkers and reviewed the efficacy of various diagnostic methods, such as synovial fluid analysis, serum markers, and molecular techniques, their results particularly suggesting the superiority of synovial fluid biomarkers over serum ones. They further highlighted the promise of diagnostic algorithms that integrate these biomarkers with other clinical and radiological parameters for a more accurate diagnosis. Despite the promising findings, further research is needed to fully validate the clinical utility of these biomarkers. The review highlights the updates of diagnostic biomarkers and provides valuable insights into their potential role in enhancing PJI diagnosis.

Determining the optimal interval for two-stage exchange in PJI remains a complex and controversial issue, as explored in the review by Sousa et al. [23]. They examined the existing evidence and proposed that an interval of 8 weeks may achieve optimal success rates. However, this interval should not be applied universally but should be tailored according to individual patient factors, including the type of infecting organism, comorbidities, and the local soft tissue condition. The authors also discussed the imaging and intraoperative findings as guides for this crucial decision-making process. Their comprehensive exploration provided valuable data for surgeons and highlights the importance of a patient-specific approach in determining the most effective interval for two-stage exchange in PJI.

### Treatment protocols and patient outcomes

There are several treatment strategies for PJI management, each having unique advantages and challenges. Gramlich et al. [24] conducted an in-depth review on salvage procedures. These procedures included implant retention with irrigation and debridement, a technique that aims to preserve the prosthesis while eliminating the infection, thus minimizing disruption to joint function. The review covered the more invasive procedures of implant exchange and two-stage exchange, which involved the removal and replacement of the infected prosthesis. Meanwhile, patient selection is very important, with the patient’s overall health, the severity of the infection, and the type of infecting organism have been taken into consideration, so a multidisciplinary approach to preoperative planning is required. Complementing Gramlich’s work, Chen et al. [25] reviewed the use of articulating spacers, devices designed to maintain joint mobility and space during the interim period between the removal of the infected prosthesis and its replacement. They discussed various designs of spacers, their indications, and the technical aspects of their implantation and removal. They argued that the correct use of these spacers could significantly enhance patient comfort and mobility during the challenging period between staged revisions for PJI. Meanwhile, Cao et al. [26] comprehensively analyzed a single-stage revision as a solution for chronic PJI following knee and hip arthroplasties. This procedure involved the removal of the infected prosthesis and immediate replacement within the same surgery, thereby reducing the need for multiple surgeries. They also provided valuable suggestions on patient selection, which takes into consideration the factors such as the type of infecting organisms, the patient's general health, and the extent of the infection.

Several studies have investigated the efficacy of different surgical strategies for the treatment of acute PJI after total knee arthroplasty. Natali et al. [27]. compared debridement, antibiotics, and implant retention (DAIR) with debridement, antibiotics, and bead insertion. Their results showed no significant difference in success rates between these two methods, indicating both are viable strategies for the treatment of PJI. In another study [28], the use of gentamycin beads or sponges however showed inferior outcomes, and their use has been discouraged in some DAIR treatment protocols. This notion of flexibility in surgical strategies is reinforced by Fokkema et al. [29], who reported an unusual case of PJI caused by *Streptobacillus moniliformis*, a pathogen typically associated with rat-bite fever. This infection was successfully managed using the DAIR, underlining the importance of considering both common and rare pathogens in the differential diagnosis of PJI. Further emphasizing the effectiveness of the DAIR method, Spangehl et al. [30]. argued for its use as a first-line treatment for acute PJI, subject to appropriate technical considerations and patient selection. They also highlighted the crucial role of early intervention and suitable antibiotic therapy in attaining the success of DAIR. Complementing these findings, Mian et al. [31] reviewed current practices in PJI debridement and revision arthroplasty, including the use of antibiotics, implant retention, and two-stage revision. They believed that early detection and proper management of PJI can enhance patient outcomes and minimize the need for revision surgery. Overall, these studies underscored the potential efficacy of different surgical strategies for the treatment of PJI and the importance of individualized treatment, early intervention, and consideration of the possibility of the various causative pathogens.

Wouthuyzen-Bakker et al. [32] discussed the critical role of antibiotics in the management of PJI. They outlined the basic principles of antimicrobial treatment, focusing on rifampicin for Gram-positive bacteria and fluoroquinolones for Gram-negative bacteria, which are the most common causes of PJI. They also discussed the importance of tailoring antibiotic regimens based on culture results and patient characteristics. In addition, this group [33] presented the Northern Infection Network for Joint Arthroplasty (NINJA) protocol for PJI treatment, which incorporates the latest evidence-based practices and a multidisciplinary approach, including diagnostic, surgical and antibiotic treatment steps. This protocol serves as a model for regions seeking to enhance PJI management, highlighting the need for a comprehensive, patient-centered, and evidence-based approach.

Lastly, a special case report by Ferry et al. [34] discussed *Coxiella burnetti* prosthetic joint infection in an immunocompromised woman. A patient underwent a protracted treatment with ofloxacin-rifampin, multiple surgeries, and a complex reconstruction. This case highlighted the challenge of managing PJI in immunocompromised patients the need for a multidisciplinary approach.

### Future directions & perspective

The management of PJI and SSI has been dynamically evolving. The challenges presented by these complications require a multidisciplinary approach and a commitment to continued research and innovation. One promising pathway is the development of novel antibiotics and their delivery systems. By conceiving, rigorously testing, and using effective antibiotics, especially those with enhanced efficacy against biofilm-producing bacteria, complete with delivery systems that improve antibiotic penetration into biofilm matrices, we could revolutionize the therapeutic practice for PJI and SSI. Additionally, intra-articular antibiotic infusion represents a promising route of administration. It can circumvent systemic circulation, reduce the risk of hepatic and renal function impairment, and provide a sufficiently high antibiotic concentration at the prosthetic infection site. However, further research with a higher level of evidence are still needed to confirm the efficacy of this administration method and its impact on pathogen resistance. At the same time, the role of biofilm research is undeniably critical. Since biofilms are significant hurdles in the way, future research endeavors should be directed at looking into the biology of biofilm formation and working out strategies to disrupt or inhibit their formation. This includes investigating the genetic and environmental factors that speed up or underlie biofilm formation and developing materials that are resistant to biofilm adherence. Furthermore, there is a need for the development of physical technologies capable of directly disrupting biofilms, such as ultrasound and radiofrequency modalities. Equally important is a comprehensive understanding of patient-specific contributors to the risk of PJI and SSI. Intensive research into the role of comorbidities, genetic predispositions, and lifestyle factors in infection could help develop targeted interventions to mitigate these risks and enable shared decision-making with the patient based on his or her comorbidities and lifestyle choices. The potential of technological advancements, such as machine learning (ML) and artificial intelligence (AI), is significant. Integration of ML and AI into the management of PJI and SSI could enhance diagnostic accuracy and improve treatment outcomes. For instance, the predictive algorithms based on patient data can better assess infection risk, and the real-time patient monitoring systems can identify/capture early signs of infection. Lastly, robust, high-quality randomized controlled trials and continuous surveillance in orthopedic registries are of paramount importance to the validation of findings from observational studies and case reports and the formulation of evidence-based guidelines for PJI and SSI management.

## Conclusion

The management of PJI and SSI continues to be a formidable task within the realm of orthopedic surgery. These infections, with their inherent complexity, present a considerable challenge, but significant progress has been made in understanding their pathophysiology, pinpointing risk factors, and developing effective countermeasures. The future of PJI and SSI management depends on sustained research, interdisciplinary efforts, and the integration of novel technologies. By constantly expanding our knowledge and honing our skills, we can improve patient outcomes and ease the burden these infections place on our healthcare systems.

## Data Availability

Not applicable.
